# Adiponectin Receptor 2 Deficiency Results in Reduced Atherosclerosis in the Brachiocephalic Artery in Apolipoprotein E Deficient Mice

**DOI:** 10.1371/journal.pone.0080330

**Published:** 2013-11-12

**Authors:** Anna Lindgren, Malin Levin, Sandra Rodrigo Blomqvist, Johannes Wikström, Andrea Ahnmark, Christina Mogensen, Gerhard Böttcher, Mohammad Bohlooly-Y, Jan Borén, Li-Ming Gan, Daniel Lindén

**Affiliations:** 1 Cardiovascular & Metabolic Disease Innovative Medicines, AstraZeneca R&D, Mölndal, Sweden; 2 Wallenberg Laboratory for Cardiovascular Research, Sahlgrenska Academy, University of Gothenburg, Gothenburg, Sweden; 3 Discovery Sciences, Transgenics, AstraZeneca R&D, Mölndal, Sweden; 4 Global Safety Assessment, Pathology Sciences, AstraZeneca R&D, Mölndal, Sweden; University of Padova, Italy

## Abstract

Adiponectin has been shown to have beneficial cardiovascular effects and to signal through the adiponectin receptors, AdipoR1 and AdipoR2. The original aim of this study was to investigate the effect of combined *AdipoR1* and *AdipoR2* deficiency (AdipoR1^-/-^AdipoR2^-/-^) on atherosclerosis. However, we made the interesting observation that *AdipoR1* ^*-/-*^ *AdipoR2* ^*-/-*^ leads to embryonic lethality demonstrating the critical importance of the adiponectin signalling system during development. We then investigated the effect of *AdipoR2*-ablation on the progression of atherosclerosis in *apolipoprotein E* deficient (*ApoE* ^*-/-*^) mice. AdipoR2^-/-^ApoE^-/-^ mice fed an atherogenic diet had decreased plaque area in the brachiocephalic artery compared with *AdipoR2* ^*+/+*^ApoE^-/-^ littermate controls as visualized *in vivo* using an ultrasound biomicroscope and confirmed by histological analyses. The decreased plaque area in the brachiocephalic artery could not be explained by plasma cholesterol levels or inflammatory status. However, accumulation of neutral lipids was decreased in peritoneal macrophages from AdipoR2^-/-^ApoE^-/-^ mice after incubation with oxidized LDL. This effect was associated with lower CD36 and higher ABCA1 mRNA levels in peritoneal macrophages from AdipoR2^-/-^ApoE^-/-^ mice compared with AdipoR2^+/+^ApoE^-/-^ controls after incubation with oxidized LDL. In summary, we show that adiponectin receptors are crucial during embryonic development and that *AdipoR2*-deficiency slows down the progression of atherosclerosis in the brachiocephalic artery of *ApoE*-deficient mice.

## Introduction

Adiponectin is one of the most abundant factors secreted from adipocytes [1–4]. Hypoadiponectinemia is observed in patients with obesity [5], type 2 diabetes [6] and coronary artery disease [7,8] and high levels of adiponectin reduce the risk of developing type 2 diabetes [9] and myocardial infarction [10]. 

Several studies in experimental animals have demonstrated anti-diabetic [11–13] and anti-atherosclerotic [12–15] effects of adiponectin. In addition, adiponectin can protect the heart from ischemia-reperfusion injury [16] and can act as an endogenous anti-thrombotic factor [17]. The anti-atherosclerotic effects of adiponectin have been demonstrated at several stages in plaque development, ranging from endothelial dysfunction and plaque initiation to plaque progression and rupture (reviewed in 18). *In vitro* studies have shown that adiponectin suppresses monocyte adhesion and expression of endothelial cell adhesion molecules, such as intracellular adhesion molecule-1 (ICAM-1), vascular cell adhesion molecule-1 (VCAM-1) and E-selectin [7]. Furthermore, adiponectin inhibits foam cell formation by downregulating macrophage scavenger receptor A, resulting in reduced lipid accumulation in macrophages [19,20]. Studies have also shown that adiponectin can induce cholesterol efflux from macrophages via upregulation of the ATP-binding cassette transporter ABCA1 [20,21]. 

Adiponectin has been proposed to mediate its effects via at least two transmembrane receptors, adiponectin receptor 1 (AdipoR1) and adiponectin receptor 2 (AdipoR2) [22]. We and others have demonstrated the physiological importance of these receptors using gene knock-out mice [23–25]. AdipoR1^-/-^ mice showed decreased glucose tolerance [23,25]. In contrast, AdipoR2^-/-^ mice were resistant to high-fat diet induced obesity and exhibited improved glucose tolerance and decreased plasma cholesterol levels [23,24]. In addition, knock-down of AdipoR2 using antisense oligonucleotides reduced plasma glucose levels in insulin resistant leptin-deficient ob/ob mice [24]. Thus, AdipoR1 and AdipoR2 are clearly involved in glucose and energy metabolism but have opposing roles [23]. 

In this study, we could show that mice lacking both *AdipoR1* and *AdipoR2* were embryonically lethal, i.e. found dead in utero at day 16.5 with macroscopical swelling and microscopical autolysis and tissue disintegration. We then hypothesized that the favourable metabolic phenotype of AdipoR2^-/-^ mice we and others have observed previously [23,24] also could be associated with a protective effect against developing atherosclerosis. We generated mice deficient in both *AdipoR2* and *ApoE* (AdipoR2^-/-^ApoE^-/-^) and littermate control mice lacking only *ApoE* (AdipoR2^+/+^ApoE^-/-^) in order to study the impact of *AdipoR2* deficiency on the atherosclerosis process. Interestingly, the progression of atherosclerosis was attenuated in the brachiocephalic artery of *AdipoR2* deficient mice.

## Methods

### Ethics Statement

All experiments were approved by the Gothenburg Ethics Committee for Experimental Animals.

### Generation of AdipoR2^-/-^ApoE^-/-^ mice

*AdipoR1* ^*-/-*^ and *AdipoR2* ^*-/-*^ mice obtained from Deltagen (San Carlos, CA) have been described previously [23]. *AdipoR2* ^*-/-*^ mice were backcrossed with C57BL/6J mice for eight generations before they were inter-crossed with ApoE^-/-^ mice (Taconic Europe, C57BL/6J). *AdipoR2* ^*+/-*^ mice were cross-bred with ApoE^-/-^ mice to generate offspring that were heterozygous for both *AdipoR2* and *ApoE* (AdipoR2^+/-^ApoE^+/-^). These heterozygous mice were then inter-crossed to generate AdipoR2^+/-^ApoE^-/-^ mice. Finally, AdipoR2^+/-^ApoE^-/-^ mice were inter-crossed to produce AdipoR2^-/-^ApoE^-/-^ mice and AdipoR2^+/+^ApoE^-/-^ littermate controls used in the study.

### Diets, body weight and termination

Male *AdipoR2* ^*-/-*^ApoE^-/-^ mice and *AdipoR2* ^*+/+*^ApoE^-/-^ littermate controls were given standard laboratory chow diet (R3; Lactamin, Kimstad, Sweden) for 8 weeks. Thereafter, they received a high-fat diet (Lard diet) containing 21% swine Lard and 0.15% cholesterol (Diet 821424; Special Diets Services, Witham, England) for 14 weeks until termination. The lard diet contained 0.03% C14:1, 0.11% C16:1, 6.69% C18:1, 1.95% C18:2, 0.11% C18:3, 0.03% C20:4, 0.03% C12:0, 0.37% C14:0, 4.56% C16:0, and 2.04% C18:0, resulting in an ω6/ ω3 ratio of 18. Body weights were registered every other week. At termination, blood was collected via heart puncture from isoflurane-anaesthetized (Forene Isoflurane, Abbott Scandinavia AB, Sweden) mice. Liver and gonadal white adipose tissue (WAT) were dissected and weighed. The cardiovascular system was perfused with NaCl, followed by dissection of the aorta and brachiocephalic artery for histological analyses. 

### Ultrasound measurements

The mice were anaesthetized with isoflurane gas and maintained anaesthetized during the ultrasound measurement with an isoflurane dose of 1.5% in room air. Ultrasound measurements were performed at 8, 15 and 22 weeks of age. Ultrasound biomicroscopy equipment (Vevo 770, Visual Sonics, Toronto, Canada) with a transducer frequency of 40 MHz was used for vascular imaging. The brachiocephalic artery was visualized as described previously [26]. 

### Blood samples, plasma and liver analyses

Blood samples were collected from Vena Saphena at 7 weeks of age (before switched to the Lard diet), at 11 and 18 weeks of age and at termination at 22 weeks of age. Lipids were extracted from plasma samples using the Folch method [27]. Triglycerides in total lipid extracts were separated using normal phase liquid chromatography, NPLC, and measured by evaporative light scattering detection, ELSD [28]. A calibration curve was obtained by injecting commercial lipid standard. Total plasma cholesterol levels were measured with an enzymatic colorimetric assay (Roche Diagnostics, Mannheim, Germany). Cholesterol distribution profiles were measured on 10 µl pooled plasma from 10 mice in each group, using a size-exclusion high performance liquid chromatography system, SMART (GE Healthcare, Waukesha, WI). Plasma adiponectin levels were measured using a radioimmunoassay from Linco Research (St. Charles, MO) and plasma cytokines were determined using bead based multiplex suspension array kits with the Luminex technology on a BioPlex (Bio-Rad, CA). Liver triglyceride content was measured on an ABX Pentra 400 (HORIBA Medical, Kyoto, Japan) after homogenization in isopropanol (1 ml/50 mg), incubation at 4°C for 1 h, and centrifugation at 2,500 rpm for 5 min.

### Histological analyses

Paraffin sections (4 µm) from the proximal brachiocephalic artery and the aortic arch were stained with Miller’s Elastin and Picrosirius Red (Histolab Products AB, Gothenburg, Sweden) and used for measurement of plaque area and collagen content, respectively. A Mac-2 antibody (CL8942AP clone M3/38; Cedarlane, Hornby, Canada) at a dilution of 1:20,000 was used for quantification of macrophages and macrophage-derived foam cells. For the en face analysis, the thoracic part of the descending aorta was cut open longitudinally and mounted in Tissue-Tek O.C.T compound (Sakura, Torrance, CA). All plaque analyses were done using BioPix iQ 2.1 Software (BioPix, Gothenburg, Sweden). Plaque area in the descending aorta is expressed as percentage of total vessel area. 

### Protein extraction and western blot

Liver protein was extracted by homogenization in T-PER Tissue Protein Extraction Reagent (Thermo Scientific, Waltham, MA) with added protease inhibitors according to the manufacturer’s protocol. Protein concentrations were determined with the Pierce BCA Protein Assay kit (Thermo Scientific). 30 µg protein was separated on a 4-12% Bis-Tris gel and subsequently transferred onto a nitrocellulose membrane. The membrane was incubated with affinity purified primary antibodies against human AdipoR2 (1 μg/ml in phosphate-buffered saline with 0.05% Tween and 5% milk powder). The AdipoR2 antibodies were generated by immunizing rabbits with the peptide CSRTPEPDIRLRKGHQLDG (94% sequence homology with the corresponding mouse sequence). The secondary HRP-conjugated goat anti-rabbit IgG antibody (Novus Biologics, Littleton, CO) was used at 1:10 000. The bands were visualized using the Novex ECL Chemiluminescent substrate reagent kit (Invitrogen, Carlsbad, CA).

### Analyses of lipid accumulation, inflammatory response and gene expression in peritoneal macrophages

Primary peritoneal macrophages were isolated by flushing the peritoneum of 8 weeks old male *AdipoR2* ^*-/-*^ *ApoE* ^*-/-*^ mice and *AdipoR2* ^*+/+*^ApoE^-/-^ littermate controls with PBS. Cells were collected and allowed to adhere to a cell culture dish for 2 h in serum-free RPMI 1640 media supplemented with sodium-pyruvate (2mM), non-essential amino acids, sodium bicarbonate (1.5 g/l), penicillin (100 U/ml) and streptomycin (100 mg/l) at 37°C. Cells not attached to the plastic after 2 h were removed by washing three times with PBS and adherent cells were cultured in supplemented RPMI 1640 media containing 10% FCS. For analysis of inflammatory response, peritoneal macrophages were incubated with or without oxidized LDL (oxLDL) (50 µg/ml) or LPS (0.1 mg/ml) for 24 h and cytokine levels in the culture medium were determined as described above. For analysis of lipid accumulation, macrophages were incubated with or without oxidized LDL (oxLDL) (50µg/ml) for 24 h, followed by staining with oil red O. Quantification of the total oil red O area was assessed using BioPix software. For gene expression analyses, total RNA was extracted from cultured macrophages using an RNeasy kit (Qiagen, Hilden, Germany) and cDNA was synthesized using a High Capacity cDNA Archive Kit (Applied Biosystems, Foster City, CA). Real-time PCR analysis was performed with an ABI Prism 7900 HT Detection System (Applied Biosystems) using the following TaqMan Gene Expression assays: Mm00446214 for scavenger receptor class A (SR-A), Mm00450236 for SR-B1, Mm00432401 for CD36, Mm0043739 for ATP-binding cassette sub-family G member 1 (ABCG1) and Mm00442646 for ABC sub-family A member 1 (ABCA1). Primers and FAM/TAMRA labelled fluorogenic probes were used for real-time PCR analysis using the following sequences: AdipoR1 Forward primer 5´-TGGCTGAAAGACAACGACTACCT-3´, Reverse primer 5´-TGAAGCA AGCCCGAAAGG-3´, Probe 5´-ACATGGCCACAG ACCACCTATGCCC-3´, interleukin-6 (IL-6) Forward primer 5´-ACACATGTTCTCTGGGAAATCGT-3´, Reverse primer 5´-AAGTGCATCATCGTTGTTCATACA-3´, Probe 5´-AAATGAGAAAAGAGTTGTGCAATGGCAATTCTG-3´, IL-10, Forward primer 5´-CCAGAGCCACATGCTCCTAGA-3´, Reverse primer 5´-GGTCCTTTGTTTGAAAGAAAGTCTTC -3´, Probe 5´-CTGCGGACTGCCTTCAGCCAGG-3´. All TaqMan Gene Expression assays, primers and probes were from Applied Biosystems. Expression data were normalized against ribosomal 18S RNA or mouse acidic ribosomal phosphoprotein P0 (m36B4). The relative expression levels were calculated according to the formula 2^-ΔCT^, where ΔCT is the difference in cycle threshold (CT) values between the target and the ribosomal 18S RNA internal control.

### Statistics

Values are expressed as means ± SEM. Comparisons between groups were made by Kruskal-Wallis test and Mann-Whitney *U* test. *P*<0.05 was considered statistically significant.

## Results

### Generation of AdipoR1 AdipoR2 double deficient (AdipoR1^-/-^AdipoR2^-/-^) mice

The generation of *AdipoR1* ^*-/-*^ and *AdipoR2* ^*-/-*^ mice and their respective phenotypes in energy and glucose homeostasis have been described before [23]. In order to study the effects of combined *AdipoR1* and *AdipoR2* deficiency on atherosclerosis during *ApoE* deficiency, we first wanted to produce *AdipoR1* ^*-/-*^ *AdipoR2* ^*-/-*^ mice. Mice heterozygous for the two receptors (*AdipoR1* ^*+/-*^ *AdipoR2* ^*+/-*^) were generated using *AdipoR1* ^*-/-*^ males and *AdipoR2* ^*-/-*^ females (backcrossed for eight generations towards C57Bl/6J). We used this strategy since *AdipoR2* ^*-/-*^ males in contrast to *AdipoR1* ^*-/-*^ males did not produce any offspring (Table 1). This is probably due to an atrophy of the seminiferous tubules and aspermia associated with reduced testes weight as we have described before [23].

**Table 1:** Litter sizes produced by *AdipoR1* ^*-/-*^ or *AdipoR2* ^*-/-*^ males.

	**Females**		
**Males**	***AdipoR1*^*+/+*^ (*n*=3)**	***AdipoR1*^*-/-*^ (*n*=4)**	***AdipoR1*^*-/-*^ (*n*=3)**
***AdipoR1*^*-/-*^ (*n*=2)**	22	n.d.	n.d.
***AdipoR1*^*-/-*^ (*n*=3)**	n.d.	27	n.d.
***AdipoR1*^*+/+*^ (*n*=3)**	n.d.	n.d.	15
	**Females**		
**Males**	***AdipoR2*^*+/+*^ (*n*=2)**	***AdipoR2*^*-/-*^ (*n*=2)**	***AdipoR2*^*-/-*^ (*n*=4)**
***AdipoR2*^*-/-*^ (*n*=2)**	0	n.d.	n.d.
***AdipoR2*^*-/-*^ (*n*=2)**	n.d.	0	n.d.
***AdipoR2*^*+/+*^ (*n*=4)**	n.d.	n.d.	20

*AdipoR1* ^*+/+*^ and *AdipoR2* ^*+/+*^= wild type mice. n.d. = not determined.

**Figure 1:**
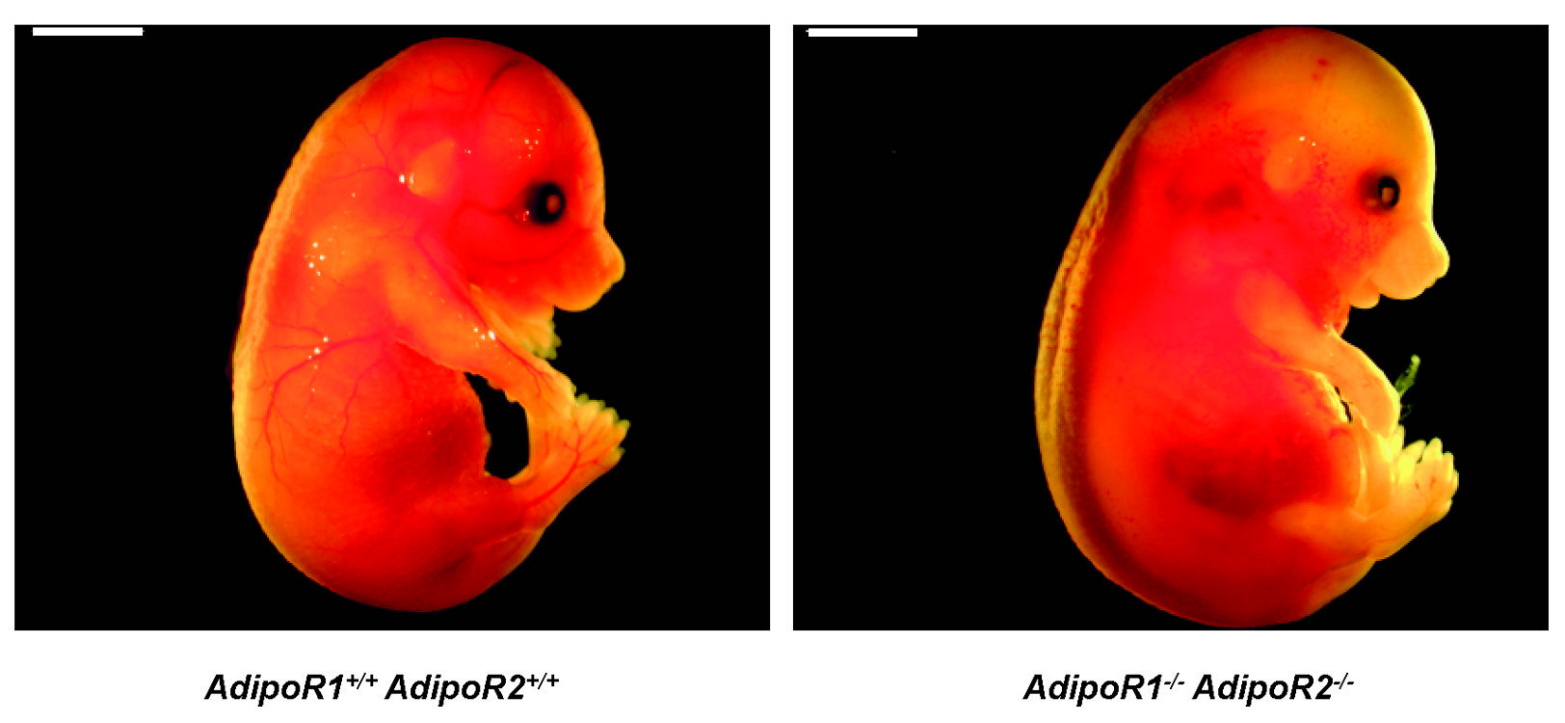
*AdipoR1* ^*+/+*^ *AdipoR2* ^*+/+*^ and *AdipoR1* ^*-/-*^ *AdipoR2* ^*-/-*^ mouse embryos at development stage 16.5 post coitum. *AdipoR1* ^*-/-*^ *AdipoR2* ^*-/-*^ mouse embryo was found dead in utero, with macroscopical swelling corresponding to the beginning autolysis and organ disintegration at the microscopical level. Scale bar: 3 mm.

Next, *AdipoR1* ^*+/-*^ *AdipoR2* ^*+/-*^ mice were inter-crossed in order to generate *AdipoR1* ^*-/-*^ *AdipoR2* ^*-/-*^ mice. Interestingly, in spite that three wild type mice were generated in this inter-cross, not a single *AdipoR1* ^*-/-*^ *AdipoR2* ^*-/-*^ mouse was detected (Table 2). In order to investigate this further, we set up another breeding using offsprings from the *AdipoR1* ^*+/-*^ *AdipoR2* ^*+/-*^ inter-cross. Male mice homozygous for *AdipoR1*-deficiency and heterozygous for *AdipoR2*-deficiency (*AdipoR1* ^*-/-*^ *AdipoR2* ^*+/-*^ mice) were bred with *AdipoR1* ^*-/-*^ *AdipoR2* ^*+/-*^, *AdipoR1* ^*+/-*^ *AdipoR2* ^*-/-*^, or *AdipoR1* ^*+/-*^ *AdipoR2* ^*+/-*^ females. Again, none of these breedings produced any *AdipoR1* ^*-/-*^ *AdipoR2* ^*-/-*^ mice (Table 3). Next, we set up an identical breeding using the same parent mice analyzing the resulting embryos at embryonic day 16.5 (Table 4). At this time point, *AdipoR1* ^*-/-*^ *AdipoR2* ^*-/-*^ mouse embryos were found dead in utero with a swollen appearance macroscopically (Figure 1), and microscopical examination at this stage showed autolysis with accompanying early disintegration of organ and tissue structures. As a specific finding we noted an intravascular accumulation of immature nucleated blood cells (data not shown). We conclude that knocking out both *AdipoR1* and *AdipoR2* in mice results in embryonic lethality.

**Table 2:** Genotypes of offspring following intercross of 4 heterozygous *AdipoR1* ^*+/-*^ *AdipoR2* ^*+/-*^ males with 8 heterozygous *AdipoR1* ^*+/-*^ *AdipoR2* ^*+/-*^ females.

	***AdipoR1^+/+^***	***AdipoR1^+/-^***	***AdipoR1^-/-^***
***AdipoR2^+/+^***	3	7	2
***AdipoR2^+/-^***	7	11	6
***AdipoR2^-/-^***	3	4	0

**Table 3:** Genotypes of offspring following intercross of *AdipoR1* ^*-/-*^ *AdipoR2* ^*+/-*^ males with *AdipoR1* ^*+/-*^ *AdipoR2* ^*+/-*^ (*n*=6) or *AdipoR1* ^*-/-*^ *AdipoR2* ^*+/-*^ (*n*=2) or *AdipoR1* ^*+/-*^ *AdipoR2* ^*-/-*^ (*n*=1) females.

	**Females**		
**Males**	***AdipoR1^+/-^AdipoR2^+/-^***	***AdipoR1^-/-^AdipoR2^+/-^***	***AdipoR1^+/-^AdipoR2^-/-^***
***AdipoR1^-/-^AdipoR2^+/-^***	6 *AdipoR1* ^*-/-*^ *AdipoR2* ^*+/+*^	7 *AdipoR1* ^*-/-*^ *AdipoR2* ^*+/-*^	1 *AdipoR1* ^*-/-*^ *AdipoR2* ^*+/-*^
	7 *AdipoR1* ^*-/-*^ *AdipoR2* ^*+/-*^	2 *AdipoR1* ^*-/-*^ *AdipoR2* ^*+/+*^	1 *AdipoR1* ^*+/-*^ *AdipoR2* ^*-/-*^
	7 *AdipoR1* ^*+/-*^ *AdipoR2* ^*-/-*^		3 *AdipoR1* ^*+/-*^ *AdipoR2* ^*+/-*^
	8 *AdipoR1* ^*+/-*^ *AdipoR2* ^*+/-*^		

**Table 4:** Genotype of E16.5 embryos following intercross of *AdipoR1* ^*+/-*^AdipoR2^+/-^ males with *AdipoR1* ^*+/-*^ *AdipoR2* ^*+/-*^ or *AdipoR1* ^*+/-*^ *AdipoR2* ^*-/-*^ females.

	**Females**	
**Males**	***AdipoR1*^*+/-*^*AdipoR2*^*+/-*^ (*n*=4)**	***AdipoR1*^*+/-*^*AdipoR2*^*+/-*^ (*n*=1)**
***AdipoR1*^*+/-*^*AdipoR2*^*+/-*^**	3 *AdipoR1* ^*+/+*^ *AdipoR2* ^*+/+*^	3 *AdipoR1* ^*+/-*^ *AdipoR2* ^*+/-*^
	14 *AdipoR1* ^*+/-*^ *AdipoR2* ^*+/-*^	1 *AdipoR1* ^*+/+*^ *AdipoR2* ^*-/-*^
	**1 *AdipoR1*^*-/-*^*AdipoR2*^*-/-*^**	1 *AdipoR1* ^*+/-*^ *AdipoR2* ^*-/-*^
	2 *AdipoR1* ^*+/+*^ *AdipoR2* ^*-/-*^	2 *AdipoR1* ^*-/-*^ *AdipoR2* ^*+/-*^
	5 *AdipoR1* ^*+/+*^ *Adipo R2* ^*+/-*^	
	5 *AdipoR1* ^*-/-*^ *AdipoR2* ^*+/+*^	
	2 *AdipoR1* ^*+/-*^ *AdipoR2* ^*-/-*^	
	1 *AdipoR1* ^*-/-*^ *AdipoR2* ^*+/-*^	

### Generation of AdipoR2 ApoE double deficient AdipoR2^-/-^ApoE^-/-^ mice

**Figure 2:**
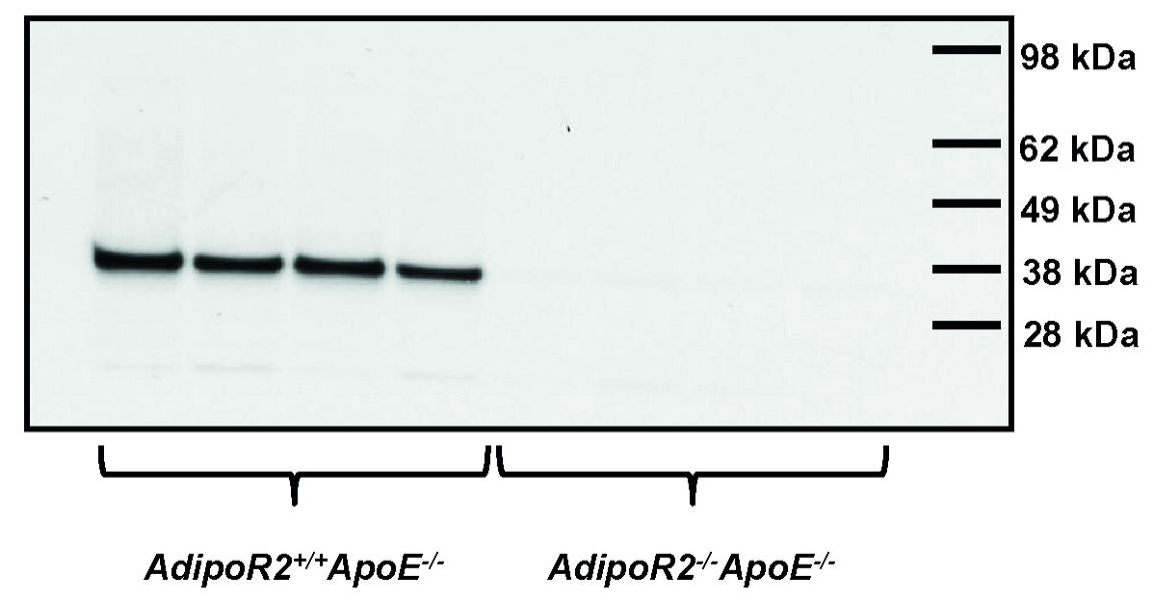
AdipoR2 protein expression in livers from *AdipoR2* ^*-/-*^ApoE^-/-^ mice and *AdipoR2* ^*+/+*^ *ApoE* ^*-/-*^ littermate controls. Absence of AdipoR2 protein in *AdipoR2* ^*-/-*^ *ApoE* ^*-/-*^ mice was confirmed by western blot analysis of liver protein extractions using affinity purified AdipoR2 antibodies (*AdipoR2* ^*+/+*^ *ApoE* ^*-/-*^ *n* = 4, *AdipoR2* ^*-/-*^ *ApoE* ^*-/-*^ *n* = 4).

Based on the previous finding by us and Liu et al [23,24] that AdipoR2^-/-^ mice are protected from high-fat diet-induced dyslipidemia and insulin resistance, we then hypothesized that *AdipoR2* deficiency could potentially have a protective role also against atherosclerosis. Mice deficient in both *AdipoR2* and *ApoE* (AdipoR2^-/-^ApoE^-/-^) and littermate control mice lacking only *ApoE* (AdipoR2^+/+^ApoE^-/-^) were generated to study the effect of *AdipoR2* deficiency on the progression of atherosclerosis. The absence of AdipoR2 protein in *AdipoR2* ^*-/-*^ *ApoE* ^*-/-*^ mice was confirmed by western blot analysis of liver protein extracts (Figure 2).

### Effect of *AdipoR2* deficiency on plaque area and morphology

**Figure 3:**
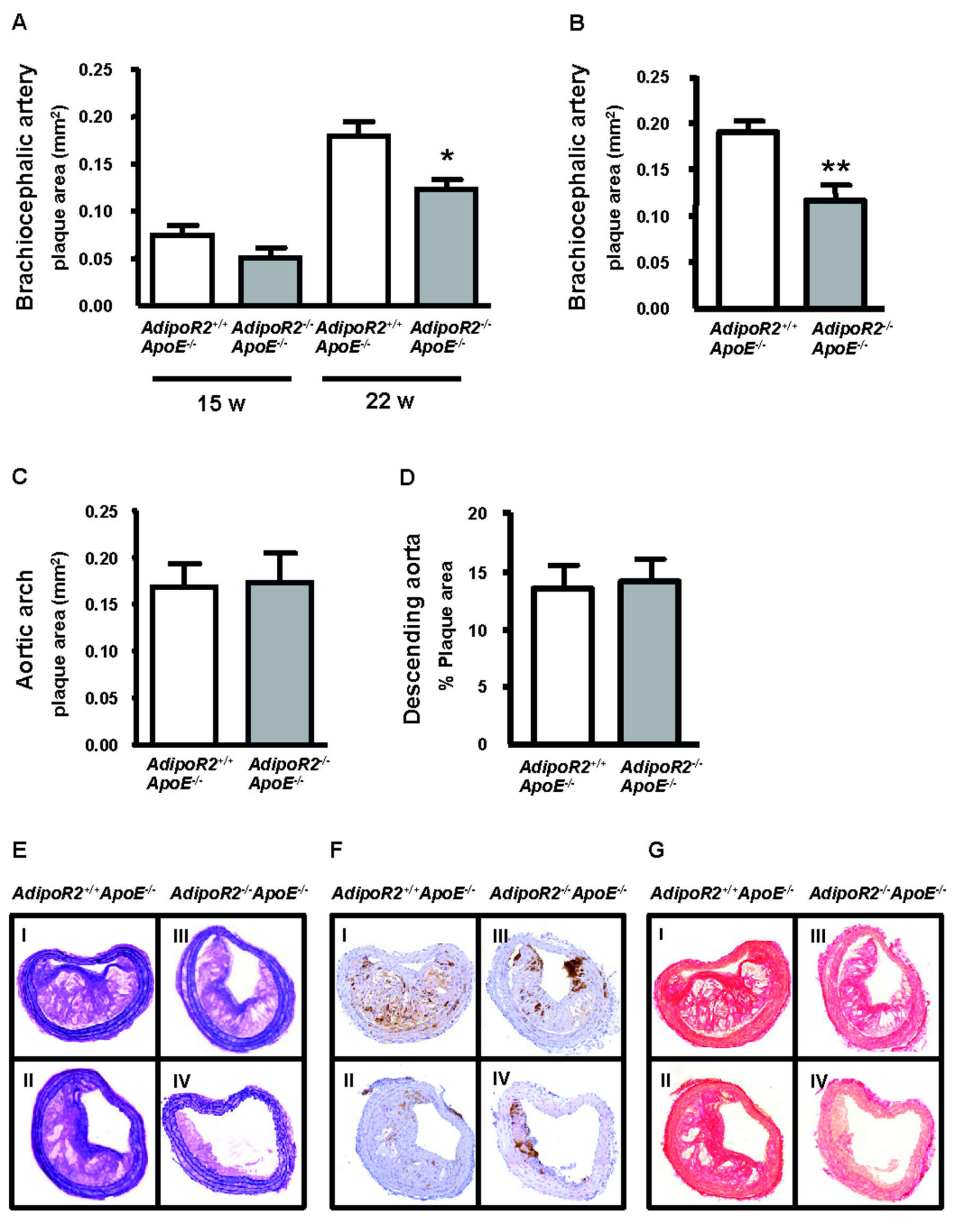
Plaque area in the brachiocephalic artery of *AdipoR2* ^*-/-*^ApoE^-/-^ mice and *AdipoR2* ^*+/+*^ *ApoE* ^*-/-*^ littermate controls after 7 and 14 weeks on Lard diet. (A) Plaque area in the brachiocephalic artery measured by ultrasound at 15 and 22 weeks of age (*AdipoR2* ^*+/+*^ *ApoE* ^*-/-*^ *n* = 25, *AdipoR2* ^*-/-*^ *ApoE* ^*-/-*^ *n* = 14). (B) Plaque area in the brachiocephalic artery measured by histology at termination at 22 weeks of age (*AdipoR2* ^*+/+*^ *ApoE* ^*-/-*^ *n* = 24, *AdipoR2* ^*-/-*^ *ApoE* ^*-/-*^ *n* = 14). (C) Plaque area in the aortic arch measured by histology at termination at 22 weeks of age (*AdipoR2* ^*+/+*^ *ApoE* ^*-/-*^ *n* = 25, *AdipoR2* ^*-/-*^ *ApoE* ^*-/-*^ *n* = 11). (D) Plaque area (% of total vessel area) in the descending aorta measured by en face analysis at termination at 22 weeks of age (*AdipoR2* ^*+/+*^ *ApoE* ^*-/-*^ *n* = 22, *AdipoR2* ^*-/-*^ *ApoE* ^*-/-*^ *n* = 14). Miller´s elastin staining (E), Mac2 staining (F), and Picrosirius Red staining (G) of representative brachiocephalic artery plaques from *AdipoR2* ^*+/+*^ApoE^-/-^ mice (I-II) and *AdipoR2* ^*-/-*^ *ApoE* ^*-/-*^ mice (III-IV). Values are means ± SEM. *P< 0.05, **P< 0.01 for comparison between *AdipoR2* ^*-/-*^ApoE^-/-^ and *AdipoR2* ^*+/+*^ApoE^-/-^ mice (Mann-Whitney *U* test).

Male *AdipoR2* ^*-/-*^ *ApoE* ^*-/-*^ mice and corresponding *AdipoR2* ^*+/+*^ *ApoE* ^*-/-*^ littermate controls were examined by ultrasound scanning to follow the progression of atherosclerotic plaques in the brachiocephalic artery. Up to 8 weeks of age, the mice were fed standard laboratory chow diet. At 8 weeks of age, no lesions could be detected by ultrasound scanning (data not shown). Thereafter the mice were fed a high-fat (Lard) diet. At 15 weeks of age (7 weeks of high-fat diet), distinct plaques were found in the brachiocephalic artery and there was a non-significant trend towards a decreased plaque burden in *AdipoR2* ^*-/-*^ *ApoE* ^*-/-*^ mice compared with *AdipoR2* ^*+/+*^ *ApoE* ^*-/-*^ littermate controls (Figure 3A). Following an additional 7 weeks on high-fat diet, the plaques had more than doubled in size. At this timepoint, *AdipoR2* ^*-/-*^ *ApoE* ^*-/-*^ mice had significantly smaller plaque area compared with *AdipoR2* ^*+/+*^ *ApoE* ^*-/-*^ littermate controls (Figure 3A). This effect on plaque area was not related to vessel size, since the diameter of the brachiocephalic artery was similar between the genotypes (*AdipoR2* ^*+/+*^ *ApoE* ^*-/-*^; 0.85 ± 0.02 mm *vs. AdipoR2* ^*-/-*^ *ApoE* ^*-/-*^; 0.83 ± 0.02 mm, *P*=0.90, Mann-Whitney *U* test). After the final ultrasound scan, all mice were terminated and the brachiocephalic arteries and descending aortas were dissected for histological analyses of plaque size and morphology. The plaque area in *AdipoR2* ^*-/-*^ *ApoE* ^*-/-*^ mice was 40% smaller compared with *AdipoR2* ^*+/+*^ *ApoE* ^*-/-*^ controls (Figure 3B). In contrast to the brachiocephalic artery, the plaque area measured by histology in the aortic arch and en face analysis of the descending aorta showed a similar plaque burden between the genotypes (Figure 3C and D). 

In addition to plaque area measurements, the histological samples were used to study plaque morphology in the brachiocephalic artery. Plaques in *AdipoR2* ^*+/+*^ApoE^-/-^ mice were generally larger and characterized by cholesterol clefts and a clear fibrous cap, whereas plaques in *AdipoR2* ^*-/-*^ApoE^-/-^ mice in most cases were smaller and contained a high degree of macrophages, although cholesterol clefts and fibrous caps were also present in some of these plaques (Figure 3E-G). The Mac2-immunopositive area (i.e. area of stained macrophages and/ or macrophage-derived foam cells) was larger in plaques from *AdipoR2* ^*-/-*^ApoE^-/-^ mice compared with *AdipoR2* ^*+/+*^ApoE^-/-^ controls (*AdipoR2* ^*+/+*^ *ApoE* ^*-/-*^; 12.5 ± 2.2 % *vs. AdipoR2* ^*-/-*^ *ApoE* ^*-/-*^; 27.1 ± 4.4 % of the total plaque area, *P*=0.002, Mann-Whitney *U* test). In contrast, picrosirius staining demonstrated a lower collagen content in plaques from *AdipoR2* ^*-/-*^ *ApoE* ^*-/-*^ mice compared with *AdipoR2* ^*+/+*^ApoE^-/-^ controls (*AdipoR2* ^*+/+*^ *ApoE* ^*-/-*^; 43.6 ± 3.2 % *vs. AdipoR2* ^*-/-*^ *ApoE* ^*-/-*^; 32.3 ± 4.1 % of the total plaque area, *P*=0.049, Mann-Whitney *U* test). No obvious calcifications were noted in any of the genotypes, thus no difference could be detected between the groups in this respect.

### Effects of *AdipoR2*-deficiency on body weight and plasma biochemistry

**Figure 4:**
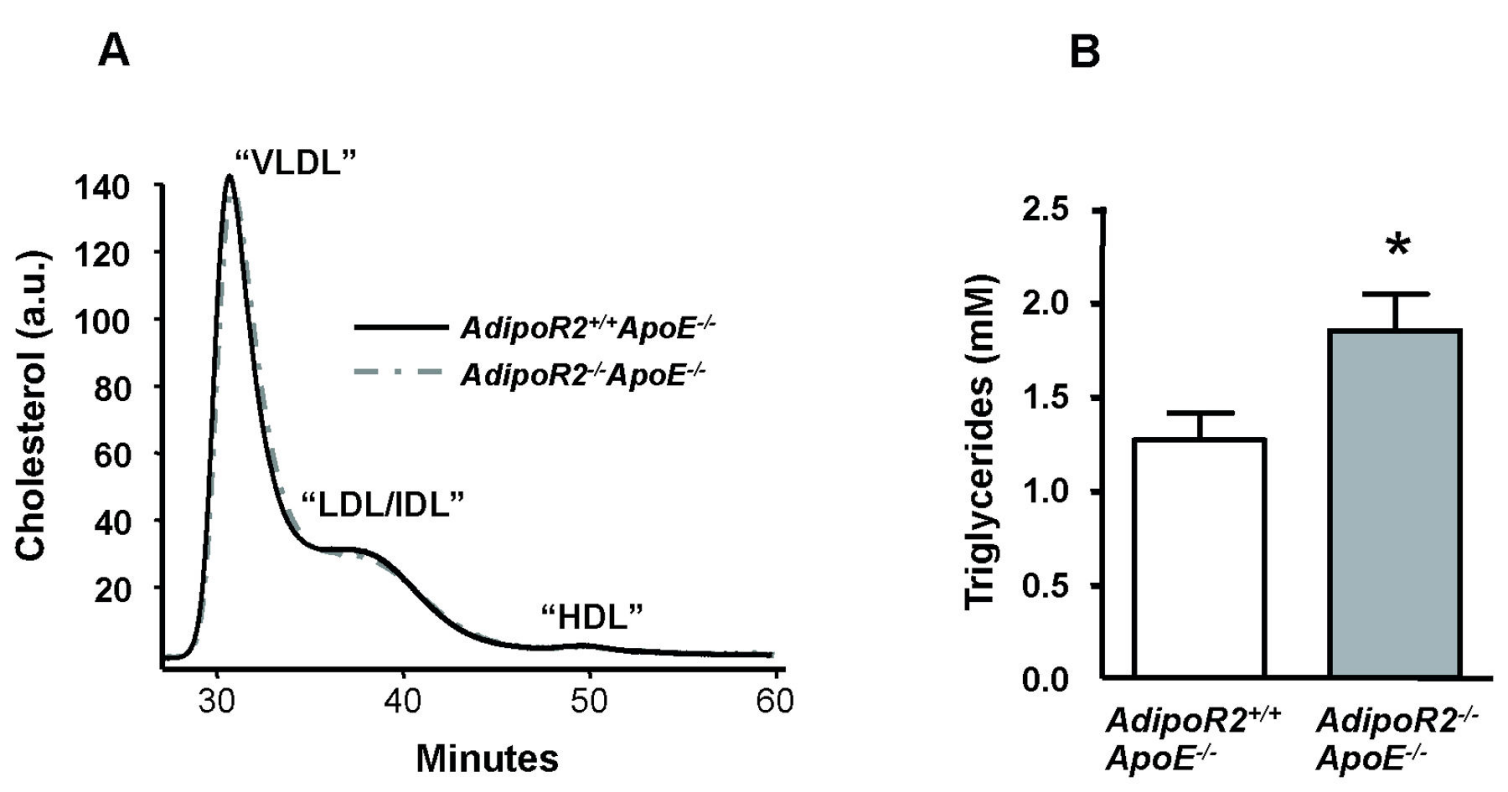
Plasma lipids in 22 weeks old *AdipoR2* ^*-/-*^ *ApoE* ^*-/-*^ mice and *AdipoR2* ^*+/+*^ *ApoE* ^*-/-*^ littermate controls after 14 weeks on Lard diet. (A) Cholesterol distribution profiles were measured by size exclusion HPLC on pooled plasma from 10 mice in each group. (B) Plasma triglycerides were measured in lipid extracts from plasma after separation by normal phase liquid chromatography, NPLC (*AdipoR2* ^*+/+*^ *ApoE* ^*-/-*^ *n* = 25, *AdipoR2* ^*-/-*^ *ApoE* ^*-/-*^ *n* = 14). Values are means ± SEM. *P< 0.05 (Mann-Whitney *U* test).

Body weights, body weight gain, WAT, liver weights and liver triglyceride content were similar between *AdipoR2* ^*-/-*^ *ApoE* ^*-/-*^ mice and *AdipoR2* ^*+/+*^ApoE^-/-^ controls (Table 5). Total cholesterol levels were followed during the study and measured at four time points (Table 6). Cholesterol levels were similar between the genotypes, except for the measurement at termination when *AdipoR2* ^*-/-*^ApoE^-/-^ mice had higher plasma cholesterol levels compared with *AdipoR2* ^*+/+*^ApoE^-/-^ controls (Table 6). Since the plasma cholesterol levels were equal, or at termination even higher in *AdipoR2* ^*-/-*^ApoE^-/-^ mice, the smaller plaque size in these mice must be explained by mechanisms other than reduction in total cholesterol levels. In addition, the cholesterol distribution profiles were almost identical between the groups (Figure 4A). Furthermore, the plasma triglyceride levels were higher at termination in *AdipoR2* ^*-/-*^ApoE^-/-^ mice compared with *AdipoR2* ^*+/+*^ApoE^-/-^ controls (Figure 4B). *AdipoR2* deficiency had no effect on total plasma adiponectin levels (Table 6). Thus, neither plasma levels of lipids or adiponectin could help to explain the protective effect of *AdipoR2* deficiency against atherosclerosis. 

**Table 5:** Body and organ weights of *AdipoR2* ^*-/-*^ *ApoE* ^*-/-*^ mice and *AdipoR2* ^*+/+*^ *ApoE* ^*-/-*^ littermate controls.

	***AdipoR2*^*+/+*^*ApoE*^*-/-*^ (*n*=25)**	***AdipoR2*^*-/-*^*ApoE*^*-/-*^ (*n*=14)**
**Body weight at termination (g)**	30.3 ± 0.63	30.8 ± 0.81
**Body weight gain (g)**	5.20 ± 0.56	5.40 ± 0.57
**Body weight gain (g/day)**	0.05 ± 0.01	0.06 ± 0.01
**Reproductive WAT (g)**	0.86 ± 0.09	0.90 ± 0.11
**Reproductive WAT (% of bw)**	2.74 ± 0.22	2.87 ± 0.27
**Liver (g)**	1.39 ± 0.04	1.34 ± 0.04
**Liver (% of bw)**	4.59 ± 0.09	4.35 ± 0.05
**Liver triglycerides (g TG/ 100g liver)**	7.6 ± 1.7 (*n* = 10)	5.1 ± 0.7 (*n* = 10)

The mice were fed Lard diet from 8 weeks of age until termination at 22 weeks of age. Values are means ± SEM.

**Table 6:** Plasma cholesterol and adiponectin levels in *AdipoR2* ^*-/-*^ *ApoE* ^*-/-*^ mice and *AdipoR2* ^*+/+*^ *ApoE* ^*-/-*^ littermate controls.

	***AdipoR2*^*+/+*^*ApoE*^*-/-*^ (*n*=25)**	***AdipoR2*^*-/-*^*ApoE*^*-/-*^ (*n*=14)**
**Cholesterol, 7 w (mmol/l)**	9.5 ± 0.35	10.6 ± 0.48
**Cholesterol, 11 w (mmol/l)**	49 ± 1.40	54 ± 3.30
**Cholesterol, 18 w (mmol/l)**	52 ± 1.40	51 ± 3.20
**Cholesterol, 22 w (mmol/l)**	36 ± 1.01	41 ± 2.03 *
**Adiponectin, 7 w (nmol/l)**	361 ± 40	308 ± 24
**Adiponectin, 11 w (nmol/l)**	360 ± 18	359 ± 20
**Adiponectin, 18 w (nmol/l)**	321 ± 23	361 ± 21
**Adiponectin, 22 w (nmol/l)**	312 ± 17	308 ± 16

The mice were fed Lard diet from 8 weeks of age until termination at 22 weeks of age. Plasma levels of cholesterol and adiponectin were measured during the study as described in Methods. Values are means ± SEM. *P< 0.05 (Mann-Whitney *U* test).

### Effects of *AdipoR2* deficiency on inflammation

Plasma levels of the inflammatory cytokines IL-1α, IL-1β, IFN-γ and TNF-α were similar between the genotypes and the plasma IL-6 levels were higher in *AdipoR2* ^*-/-*^ApoE^-/-^ mice compared with *AdipoR2* ^*+/+*^ApoE^-/-^ controls (Table 7). Interestingly, adiponectin treatment has been shown to enhance insulin sensitivity by increasing IRS-2 expression in the liver via an IL-6 dependent pathway [29]. However, liver IRS-2 mRNA levels were similar between the genotypes (*AdipoR2* ^*+/+*^ApoE^-/-^ = 100±27 *vs. AdipoR2* ^*-/-*^ApoE^-/-^ = 65±21 % of control, *n* = 10, *P*=0.2, Mann-Whitney *U* test) indicating that the elevated IL-6 levels observed in *AdipoR2* ^*-/-*^ApoE^-/-^ mice was not sufficient to drive an enhanced IRS-2 expression in the liver.

**Table 7:** Plasma levels of inflammatory mediators in *AdipoR2* ^*-/-*^ *ApoE* ^*-/-*^ mice and *AdipoR2* ^*+/+*^ *ApoE* ^*-/-*^ littermate controls.

	***AdipoR2*^*+/+*^*ApoE*^*-/-*^ (*n*=25)**	***AdipoR2*^*-/-*^*ApoE*^*-/-*^ (*n*=14)**
**IL-1α (pg/ml)**	19 ± 2.1	20 ± 1.7
**IL-1β (pg/ml)**	226 ± 49	245 ± 35
**IFN-γ (pg/ml)**	87 ± 13	108 ± 49
**TNF-α (pg/ml)**	488 ± 215	373 ± 52
**IL-6 (pg/ml)**	81 ± 12	122 ± 21*

The mice were fed Lard diet from 8 weeks of age until termination at 22 weeks of age. Plasma levels of inflammatory mediators were measured at termination as described in Methods. Values are means ± SEM. *P< 0.05 (Mann-Whitney *U* test).

To investigate if there was a different inflammatory response in macrophages from *AdipoR2* ^*-/-*^ApoE^-/-^ mice and *AdipoR2* ^*+/+*^ApoE^-/-^ controls, peritoneal macrophages were incubated with or without oxLDL or LPS for 24 h and the secretion of IL-1α, IL-1β, IL-6 and TNF-α into the medium was analyzed. As expected, LPS induced a strong inflammatory response, but there was no difference in the response between the genotypes (Table 8). Incubation of *AdipoR2* ^*-/-*^ApoE^-/-^ macrophages with oxLDL increased the level of IL-1α in the culture medium whereas this was not seen in culture medium from *AdipoR2* ^*+/+*^ApoE^-/-^ control macrophages (Table 8). Thus, the decreased progression of atherosclerosis in *AdipoR2* ^*-/-*^ApoE^-/-^ mice was likely due to other mechanisms than decreased inflammation. 

**Table 8:** Secretion of inflammatory mediators from peritoneal macrophages isolated from *AdipoR2* ^*-/-*^ *ApoE* ^*-/-*^ mice and *AdipoR2* ^*+/+*^ *ApoE* ^*-/-*^ littermate controls.

	**Stimuli**	***AdipoR2*^*+/+*^*ApoE*^*-/-*^ (*n*=7-9)**	***AdipoR2*^*-/-*^*ApoE*^*-/-*^ (*n*=7-8)**
**IL-1α (pg/ml)**	Basal	219 ± 47	162 ± 52
	oxLDL	307 ± 58	512 ± 165^*^
	LPS	663 ± 66^**^	633 ± 102^**^
**IL-1β (pg/ml)**	Basal	15 ± 0.7	21 ± 4.7
	oxLDL	15 ± 0.7	31 ± 9.3
	LPS	228 ± 48^***^	215 ± 43^**^
**TNF-α (pg/ml)**	Basal	15 ± 1.4	19 ± 2.7
	oxLDL	13 ± 1.4	105 ± 90
	LPS	2852 ± 440^***^	2938 ± 823^***^
**IL-6 (pg/ml)**	Basal	1247 ± 251	2224 ± 633
	oxLDL	807 ± 187	2451 ± 1457
	LPS	20555 ± 2864^***^	24759 ± 4480^***^

Peritoneal macrophages were isolated from male *AdipoR2-/-ApoE* ^*-/-*^ mice and AdipoR2^+/+^ApoE^-/-^ littermate controls, and cultured in supplemented RPMI 1640 media containing 10% FCS. Inflammatory mediators in the culture medium were analyzed after incubation with or without oxidized LDL (oxLDL) (50µg/ml) or LPS (0.1 mg/ml) for 24 h. Values are means ± SEM. ^*^ *P*< 0.05, ^**^ *P*< 0.01, ^***^ *P*< 0.001 for comparison between oxLDL or LPS *vs.* Basal within each genotype (Kruskal Wallis test followed by Mann-Whitney *U* test).

### Effects of *AdipoR2* deficiency on lipid accumulation and gene expression in peritoneal macrophages

To investigate if there was a difference in lipid accumulation, primary peritoneal macrophages were incubated with or without oxLDL for 24 h and the lipid accumulation was assessed with oil red O staining. AdipoR1 mRNA was expressed at similar levels in macrophages from both *AdipoR2* ^*-/-*^ApoE^-/-^ and *AdipoR2* ^*+/+*^ *ApoE* ^*-/-*^ mice and there was no change in AdipoR1 mRNA levels after exposure to oxLDL (data not shown).

**Figure 5:**
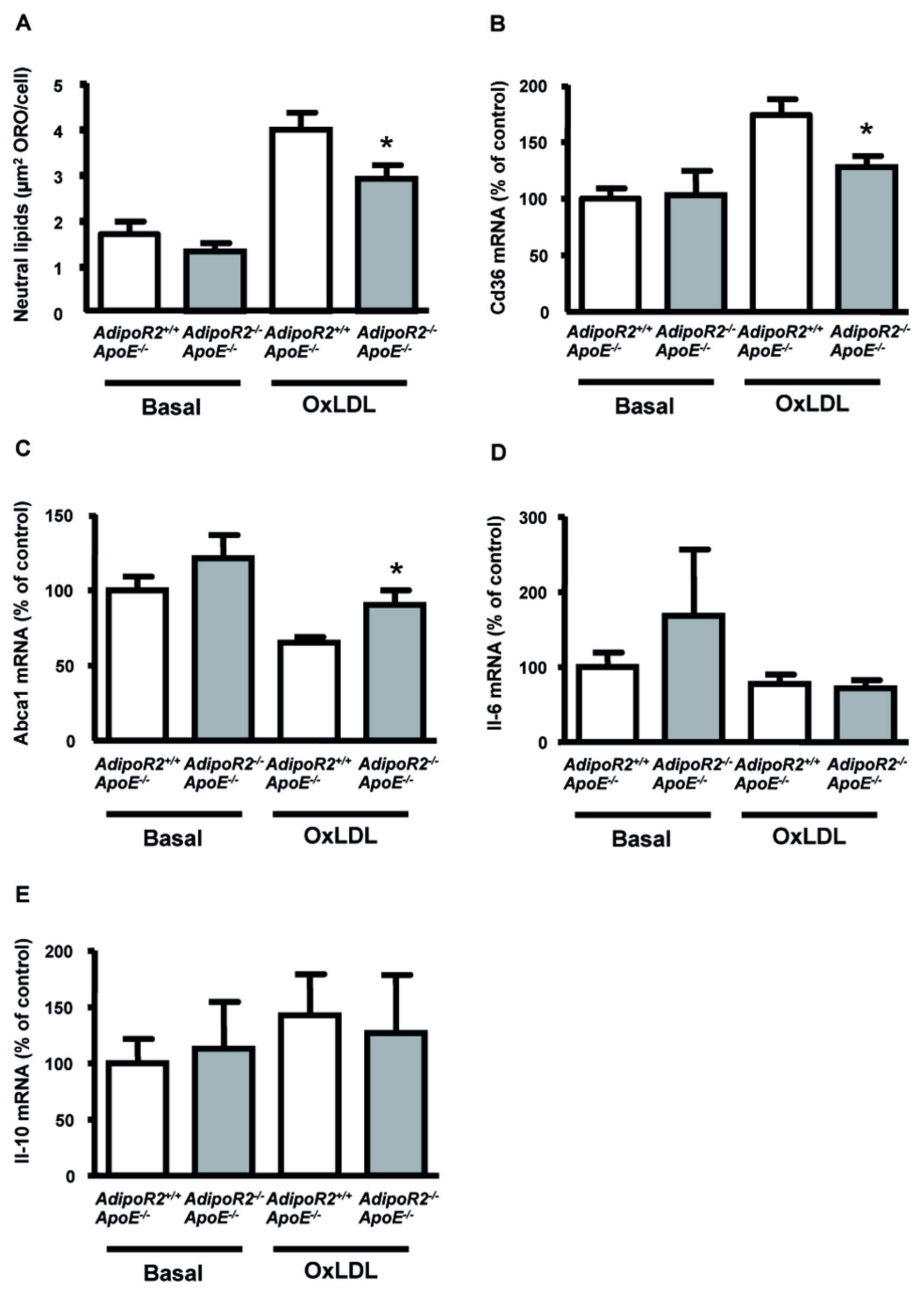
Lipid accumulation and gene expression in primary macrophages from 8 weeks old *AdipoR2* ^*-/-*^ *ApoE* ^*-/-*^ mice and *AdipoR2* ^*+/+*^ *ApoE* ^*-/-*^ littermate controls. (A) Lipid accumulation in primary macrophages was analyzed after incubation with or without 50 μg/ml oxLDL for 24 h (Basal; *AdipoR2* ^*+/+*^ *ApoE* ^*-/-*^ *n* = 18, *AdipoR2* ^*-/-*^ *ApoE* ^*-/-*^ *n* = 15. OxLDL; *AdipoR2* ^*+/+*^ *ApoE* ^*-/-*^ *n* = 14, *AdipoR2* ^*-/-*^ *ApoE* ^*-/-*^ *n* = 13). Lipid accumulation is expressed as total oil red O area per cell. (B) CD36, (C) ABCA1, (D) IL-6, and (E) IL-10 mRNA expression in primary macrophages incubated with or without 50 μg/ml oxLDL for 24 h (Basal; *AdipoR2* ^*+/+*^ *ApoE* ^*-/-*^ *n* = 8, *AdipoR2* ^*-/-*^ *ApoE* ^*-/-*^ *n* = 6. OxLDL; *AdipoR2* ^*+/+*^ *ApoE* ^*-/-*^ *n* = 8, *AdipoR2* ^*-/-*^ *ApoE* ^*-/-*^ *n* = 5). Values are means ± SEM. *P< 0.05 for comparison between *AdipoR2* ^*-/-*^ApoE^-/-^ and *AdipoR2* ^*+/+*^ApoE^-/-^ macrophages within each treatment group (Mann-Whitney *U* test).

Interestingly, accumulation of neutral lipids was decreased by 27% in macrophages from *AdipoR2* ^*-/-*^ApoE^-/-^ mice compared with macrophages from *AdipoR2* ^*+/+*^ *ApoE* ^*-/-*^ controls after incubation with oxLDL (Figure 5A). In order to explain the decreased lipid accumulation in macrophages from *AdipoR2* ^*-/-*^ApoE^-/-^ mice, gene expression levels for the scavenger receptors CD36, SR-B1 and SR-A1, and the cholesterol transporters ABCA1 and ABCG1 were measured in peritoneal macrophages incubated with or without oxLDL for 24 h. The expression levels of SR-A1, SR-B1 and ABCG1 mRNA were similar between the genotypes (data not shown). However, CD36 mRNA was downregulated by 26% in oxLDL treated macrophages from *AdipoR2* ^*-/-*^ApoE^-/-^ mice compared with control macrophages (Figure 5B), which may indicate a decreased lipid uptake. Furthermore, ABCA1 mRNA was upregulated by 39% in oxLDL treated macrophages from *AdipoR2* ^*-/-*^ApoE^-/-^ mice compared with control macrophages (Figure 5C), which could result in increased lipid efflux. These data may help to explain the reduced accumulation of lipids in macrophages from *AdipoR2* ^*-/-*^ApoE^-/-^ mice compared with macrophages from *AdipoR2* ^*+/+*^ *ApoE* ^*-/-*^ littermate controls. Finally, mRNA expression levels of interleukin (IL)-6 (Figure 5D) and IL-10 (Figure 5E) were similar in macrophages obtained from *AdipoR2* ^*-/-*^ApoE^-/-^ mice and *AdipoR2* ^*+/+*^ *ApoE* ^*-/-*^ littermate controls indicating that AdipoR2-deficiency did not affect the activation pattern (classically activated versus alternatively activated) of the macrophages.

## Discussion

In this study in male mice, we could for the first time show that knocking out both *AdipoR1* and *AdipoR2* results in embryonic lethality demonstrating the critical importance of these receptors and the adiponectin signalling system during embryonic development. We could also demonstrate the novel finding that *AdipoR2* deficiency has a protective effect on the progression of atherosclerosis in *ApoE* ^*-/-*^ mice. *AdipoR2* ^*-/-*^ *ApoE* ^*-/-*^ mice had smaller plaque area in the brachiocephalic artery compared with *AdipoR2* ^*+/+*^ *ApoE* ^*-/-*^ littermate controls. The protective effect was unrelated to changes in plasma cholesterol levels, inflammatory status or differences in the distribution of cholesterol in lipoprotein fractions. However, the protective effect of *AdipoR2* deficiency in the brachiocephalic artery could be associated with decreased lipid accumulation in macrophages as demonstrated in intraperitoneal macrophages upon incubation with oxLDL.

At embryonic day 16.5, an *AdipoR1* ^*-/-*^ *AdipoR2* ^*-/-*^ embryo was found dead in utero with macroscopical swelling and microscopical autolysis and tissue disintegration and intravascular accumulation of immature nucleated blood cells. Interestingly, adiponectin has been shown to stimulate angiogenesis both *in vitro* and *in vivo* [30,31], and it would be of interest for future studies to explore the contribution of the two adiponectin receptors in this process. Our finding that *AdipoR1* ^*-/-*^ *AdipoR2* ^*-/-*^ mice die during embryonic development is in contrast to a study by Yamauchi et al [25] in which viable *AdipoR1* ^*-/-*^ *AdipoR2* ^*-/-*^ mice were generated. The AdipoR2^-/-^ mice described by Yamauchi et al [25] was shown to express an aberrantly spliced AdipoR2 mRNA and the *AdipoR2* ^*-/-*^ mice studied by us and Liu et al has been shown to express exon 3 and exon 7 [24] surrounding the deleted exon 5 (corresponding to several transmembrane parts of this seven membrane receptor). Importantly, using an antibody directed against a peptide sequence in exon 2 containing the ATG (outside the targeting construct), we could confirm lack of AdipoR2 protein in livers of *AdipoR2* ^*-/-*^ *ApoE* ^*-/-*^ mice in this study and in AdipoR2^-/-^ mice in our previous study [23], indicating that complete lack of adiponectin receptors results in embryonic lethality. However, the non viable *AdipoR1* ^*-/-*^ *AdipoR2* ^*-/-*^ embryos generated in this study were on a C57Bl/6J genetic background while the *AdipoR1* ^*-/-*^ *AdipoR2* ^*-/-*^ mice generated by Yamauchi et al were on a more mixed 129/Sv x C57Bl/6 genetic background [25]. This may be the reason for the different results concerning generation of viable double receptor deficient mice between our laboratories. 

The difference in plaque area between *AdipoR2* ^*-/-*^ *ApoE* ^*-/-*^ mice and *AdipoR2* ^*+/+*^ *ApoE* ^*-/-*^ controls was seen in the brachiocephalic artery but not in the aortic arch or the descending aorta at the time of investigation. However, since the descending aorta was only analyzed using en face analysis and not cross-sectional analysis, we cannot rule out that also the descending aorta may have been affected. Site specific effects on atherosclerosis have been described previously in *ApoE* ^*-/-*^ mice in response to various compounds or genetic modifications [32–34]. This site specificity is thought to be related to differences in hemodynamic flow patterns, with sites of low shear stress, oscillatory flow or turbulent flow (e.g. at curvatures and branching points) being prone to develop atherosclerosis, whereas areas of laminar flow are more resistant [33]. The brachiocephalic artery (innominate artery) is the first branch in the aortic arch and constitutes a high-susceptibility site, where advanced lesions develop [33,35–37]. Thus, the fact that there is a difference in plaque area between the genotypes in the brachiocephalic artery but not in the arotic arch or the descending aorta in the present study could be due to the length of the study but also indicate a mild overall protective effect of *AdipoR2* deficiency against atherosclerosis. Adiponectin levels were found not to correlate with a suppression of the atherosclerosis process in mice using *adiponectin* deficient and overexpressing mice crossed with either *low-density lipoprotein receptor* or *ApoE* deficient mice [38]. AdipoR1 has been found to bind globular adiponectin with higher affinity than full-length adiponectin, while AdipoR2 seems to be an intermediate receptor [22]. Recently, AdipoR1 and AdipoR2 were found to form homo- and heteromeric complexes, with different interaction behaviours and signalling properties. Heteromers dissociated faster than homodimers in response to adiponectin binding and importantly, phosphorylation of AMP-activated protein kinase was delayed in response to adiponectin treatment in cells where heteromers were favoured [39]. It could therefore be speculated that lack of adiponectin receptor heteromers in the absence of AdipoR2 may have led to an improved AdipoR1 signalling. Thus, considering the results from the present study, AdipoR1 and AdipoR2 signalling may have opposing effects in atherosclerosis and it will be important for the future to investigate the effect of *AdipoR1* deficiency on the atherosclerosis process. 

Plaques in the brachiocephalic artery from *AdipoR2* ^*-/-*^ApoE^-/-^ mice were generally smaller and often rich in macrophages, whereas the majority of the plaques from *AdipoR2* ^*+/+*^ApoE^-/-^ mice were larger and characterized by cholesterol clefts and a clear fibrous cap. Plaques from *AdipoR2* ^*-/-*^ApoE^-/-^ mice contained less collagen and more macrophages compared with *AdipoR2* ^*+/+*^ApoE^-/-^ controls. Therefore, it cannot be ruled out that plaque stability was affected. However, macrophage infiltration and foam cell formation is an early process in plaque development, whereas extracellular matrix, including collagen, is produced in more mature lesions [40]. Thus, these compositional data indicate that plaques in *AdipoR2* ^*-/-*^ApoE^-/-^ mice represents earlier lesions with a higher content of macrophages, whereas more mature lesions are seen in the *AdipoR2* ^*+/+*^ *ApoE* ^*-/-*^ control mice. 

We and Liu et al have previously shown that AdipoR2^-/-^ mice are resistant to high-fat diet induced obesity [23,24], lasting at least until 15 weeks of high-fat diet [23]. The fact that *AdipoR2* ^*-/-*^ *ApoE* ^*-/-*^ mice and *AdipoR2* ^*+/+*^ *ApoE* ^*-/-*^ controls were comparable in terms of body weight gain or obesity in this study is however not surprising since *ApoE*-deficiency has been shown to attenuate diet-induced obesity in mice [41]. Further, we and Liu et al have shown that *AdipoR2* deficiency leads to hypocholesterolemia [23,24]. AdipoR2^-/-^ male mice have a plasma cholesterol level of ~3 mmol/l [23], while *AdipoR2* ^*-/-*^ *ApoE* ^*-/-*^ males have a plasma cholesterol level of ~40 mmol/l when fed a high-fat diet. Thus, the severe hypercholesterolemia in *ApoE* ^*-/-*^ mice attenuated the hypocholesterolemic phenotype seen in *AdipoR2* ^*-/-*^ mice [23,24]. Therefore, the decreased atherosclerosis in *AdipoR2* ^*-/-*^ *ApoE* ^*-/-*^ mice compared with *AdipoR2* ^*+/+*^ *ApoE* ^*-/-*^ controls must be explained by other mechanisms than reduced plasma cholesterol levels. Indeed, *AdipoR2* ^*-/-*^ *ApoE* ^*-/-*^ mice had even higher plasma cholesterol levels compared with control mice at termination. Although high-fat diet fed *ApoE* ^*-/-*^ mice are regularly used in preclinical atherosclerosis studies, the cholesterol levels obtained are extremely high. Thus, it will be important for future studies to investigate the long-term consequences of AdipoR2-deficiency on atherosclerosis under more moderately elevated cholesterol levels.

Since macrophages play an important role in atherogenesis as modulators of both lipid metabolism and immune responses, we isolated primary macrophages to study whether there was a difference in their capacity to accumulate lipids or secrete inflammatory mediators. The inflammatory response was similar in macrophages from *AdipoR2* ^*-/-*^ *ApoE* ^*-/-*^ mice and *AdipoR2* ^*+/+*^ *ApoE* ^*-/-*^ controls. On the other hand, primary macrophages from *AdipoR2* ^*-/-*^ *ApoE* ^*-/-*^ mice accumulated less neutral lipids compared with macrophages from *AdipoR2* ^*+/+*^ApoE^-/-^ mice upon incubation with oxLDL. Interestingly, macrophages from *AdipoR2* ^*-/-*^ *ApoE* ^*-/-*^ mice had lower expression levels of CD36 mRNA and higher expression levels of ABCA1 mRNA compared with control macrophages. Studies have demonstrated that CD36-deficiency dramatically inhibits lesion development in *ApoE* ^*-/-*^ mice [42,43], and can also have a protective effect in late stages of atherosclerosis [44]. The protective effect of CD36-deficiency was shown to be macrophage-dependent, since mice lacking CD36 selectively in macrophages were protected against atherosclerosis, whilst re-introduction of CD36 expressing macrophages resulted in an increased lesion area [45]. Furthermore, macrophage-specific overexpression of ABCA1 inhibits the progression of atherosclerosis [46], whereas macrophage-specific inactivation of ABCA1 increased foam cell formation and atherosclerosis [47]. Thus, downregulation of CD36 and upregulation of ABCA1 in macrophages may have contributed to the slower progression of atherosclerosis in the brachiocephalic artery of *AdipoR2* ^*-/-*^ *ApoE* ^*-/-*^ mice in the present study. 

Interestingly, homozygotes and heterozygotes of the *ADIPOR2* gene polymorphism rs767870 major allele were recently shown to have lower flow mediated dilation, increased intima-media thickness and increased ADIPOR2 protein levels in peripheral monocytes compared with homozygotes of the minor allele [48]. The same gene polymorphism has previously been associated with elevated liver fat content and fasting triglyceride levels [49] and type 2 diabetes [50]. Thus, high ADIPOR2 levels may be associated with an increased risk of developing cardiovascular disease in humans. 

In summary, we could for the first time show that combined genetic deletion of both adiponectin receptors results in embryonic lethality demonstrating the crucial importance of the adiponectin signalling system during embryonic development. We could also show that genetic ablation of AdipoR2 slows down the progression of atherosclerosis in the brachiocephalic artery in atherosclerosis prone male mice lacking ApoE indicating that antagonizing AdipoR2 or activating AdipoR1 may be an attractive therapeutic approach for the treatment of atherosclerosis.
